# The oxytocin receptor represents a key hub in the GPCR heteroreceptor network: potential relevance for brain and behavior

**DOI:** 10.3389/fnmol.2022.1055344

**Published:** 2022-12-08

**Authors:** Dasiel O. Borroto-Escuela, Cristina Cuesta-Marti, Alexander Lopez-Salas, Barbara Chruścicka-Smaga, Minerva Crespo-Ramírez, Emiliano Tesoro-Cruz, Daniel A. Palacios-Lagunas, Miguel Perez de la Mora, Harriët Schellekens, Kjell Fuxe

**Affiliations:** ^1^Department of Neuroscience, Karolinska Institutet, Stockholm, Sweden; ^2^Receptomics and Brain Disorders Lab, Department of Human Physiology, Faculty of Medicine, University of Malaga, Málaga, Spain; ^3^Department of Biomolecular Science, Section of Morphology, Physiology and Environmental Biology, University of Urbino, Urbino, Italy; ^4^APC Microbiome Ireland, University College Cork Cork, Ireland; ^5^Department of Anatomy and Neuroscience, University College Cork, Cork, Ireland; ^6^Instituto de Fisiología Celular, Universidad Nacional Autónoma de México, Mexico City, Mexico; ^7^Unidad de Investigación Biomédica en Inmunología e Infectología, Hospital de Infectología, Centro Médico Nacional La Raza, IMSS, Ciudad de México, Mexico

**Keywords:** G protein-coupled receptors, oligomerization, heteroreceptor complexes, oxytocin, dopamine, ghrelin, serotonin

## Abstract

In the last 10 years, it has become increasingly clear that large numbers of axon collaterals extend from the oxytocin (OXT) hypothalamic axons, especially the parvocellular components, to other brain regions. Consequently, the OXT signaling system forms, like other monoamine axons, a rich functional network across several brain regions. In this manuscript, we review the recently indicated higher order G-protein coupled heteroreceptor complexes of the oxytocin receptor (OXTR), and how these, *via* allosteric receptor-receptor interactions modulate the recognition, signaling, and trafficking of the participating receptor protomers and their potential impact for brain and behavior. The major focus will be on complexes of the OXTR protomer with the dopamine D2 receptor (D2R) protomer and the serotonin 2A (5-HT2AR) and 2C (5-HT2CR) receptor protomers. Specifically, the existence of D2R-OXTR heterocomplexes in the nucleus accumbens and the caudate putamen of rats has led to a postulated function for this heteromer in social behavior. Next, a physical interaction between OXTRs and the growth hormone secretagogue or ghrelin receptor (GHS-R1a) was demonstrated, which consequently was able to attenuate OXTR-mediated Gαq signaling. This highlights the potential of ghrelin-targeted therapies to modulate oxytocinergic signaling with relevance for appetite regulation, anxiety, depression, and schizophrenia. Similarly, evidence for 5-HT2AR-OXTR heteromerization in the pyramidal cell layer of CA2 and CA3 in the dorsal hippocampus and in the nucleus accumbens shell was demonstrated. This complex may offer new strategies for the treatment of both mental disease and social behavior. Finally, the 5-HT2CR-OXTR heterocomplexes were demonstrated in the CA1, CA2, and CA3 regions of the dorsal hippocampus. Future work should be done to investigate the precise functional consequence of region-specific OXTR heteromerization in the brain, as well across the periphery, and whether the integration of neuronal signals in the brain may also involve higher order OXTR-GHS-R1a heteroreceptor complexes including the dopamine (DA), noradrenaline (NA) or serotonin (5-HT) receptor protomers or other types of G-protein coupled receptors (GPCRs).

## Introduction

Oxytocin (OXT) is classically described as a nona-neuropeptide released from nerve terminals in the posterior pituitary into the blood circulation to control parturition uterine contractions, social bonding, and nursing milk letdown (Jurek and Neumann, 2018). This OXT neuropeptide in the posterior pituitary is mainly synthesized in the magno- and parvocellular paraventricular hypothalamic neurons and in the supraoptic neurons (Onaka and Takayanagi, 2019). It should be noted that species differences exist, e.g., in voles OXT is also expressed in the bed nucleus of the stria terminalis (Ross and Young, 2009) and, in glutamatergic neurons of the human prefrontal cortex (Zhong et al., 2022). The oxytocin neuropeptide binds its receptor the oxytocin receptor (OXTR), a G-protein coupled receptor (GPCR), expressed in the periphery and central nervous system (Jurek and Neumann, 2018; Froemke and Young, 2021). Over the last decades, it has become increasingly clear that the hypothalamic oxytocin neurons send projections and especially axon collaterals into many brain areas possessing oxytocin receptors (OXTR), suggesting its function extends to complex behaviors, including the production of food intake and social and emotional behaviors (Jurek and Neumann, 2018; Froemke and Young, 2021). Oxytocin signaling involves among other areas, the limbic system, the central and basolateral amygdala, the midbrain serotonin neurons, and other regions of the lower brain stem also including projections into the spinal cord (Eliava et al., 2016). Moreover, extrahypothalamic regions have also been shown to possess oxytocin neurons (Knobloch and Grinevich, 2014). Overall, oxytocin neurotransmission operates *via* volume transmission similar to other neuropeptides (Borroto-Escuela et al., 2015a), and secreted oxytocin in the blood circulation functions as a peptide hormone.

An important integrative molecular mechanism in the cellular plasma membrane is represented by GPCR heteroreceptor complexes (dimers or higher order complexes) that *via* allosteric receptor-receptor interactions modulate the recognition, signaling, and trafficking of the participating receptor protomers with an impact on other participating proteins (Fuxe et al., 2010a; 2014; Borroto-Escuela et al., 2014, 2015b, 2017; Fuxe and Borroto-Escuela, 2016).

In this review, we will deal with the discovered OXTR heteroreceptor complexes and their function, in which the dopamine D2R protomers (Romero-Fernandez et al., 2013; de la Mora et al., 2016), 5-HT2AR and 5-HT2CR protomers (Chruścicka et al., 2019, 2021; Wallace Fitzsimons et al., 2019) participate and discuss their potential relevance in brain and behavior. They will give a major contribution to the oxytocin field since through the allosteric receptor-receptor interactions in the OXTR heteroreceptor complexes the OXTR protomer can modulate and be modulated y the other participating receptor protomers like the D2R, 5-HT2AR, and 5-HT2CR. The integrative activity of the OXTR becomes substantially enlarged with modulation among others of the monoamine receptors.

## Neuroanatomy of The Oxytocin Pathways and Their Axon Collaterals

### Oxytocin positive neurons

The oxytocin immune-reactive nerve cell bodies only exist in the magnocellular and parvocellular areas of the paraventricular hypothalamic nucleus and in the supraoptic nucleus except for the bed nucleus of the stria terminalis (Liao et al., 2020; Froemke and Young, 2021). Most of the oxytocin positive axons in the magnocellular pathway pass *via* the median eminence into the posterior pituitary where the oxytocin nerve terminals release oxytocin into the blood capillaries to act as hormones. However, the oxytocin peptide is also released from cell bodies and dendrites into the extracellular space to act *via* volume transmission (Fuxe et al., 2010b; Borroto-Escuela et al., 2015a) to synchronize the firing of the oxytocin networks in the hypothalamus and favor waves of oxytocin release into the blood as demanded in nursing and parturition (Froemke and Young, 2021).

In the last 10 years, it has become established that a rich network of axon collaterals is formed from the oxytocin hypothalamic axons, especially the parvocellular ones, which project to different brain regions, including nucleus accumbens and caudate putamen (Jurek and Neumann, 2018; Froemke and Young, 2021). The oxytocin collaterals form rich networks in the brain, like those from the monoamine axons do (Fuxe, 1965a,b), in many areas of the brain like the hippocampus, limbic regions, dorsal and ventral striatum, amygdala, the lower brain stem and the spinal cord (Onaka and Takayanagi, 2019; Froemke and Young, 2021). The introduction of using oxytocin-Cre mice in which the Cre recombinase is expressed under the oxytocin promotor combined with oxytocin nerve cell-specific viruses carrying a Cre-dependent promotor for, e.g., a fluorescent protein, allowing for the necessary sensitivity for proper mapping of the widespread distribution of oxytocin and its receptors (Froemke and Young, 2021).

### Oxytocin receptors in the brain

The areas receiving oxytocin nerve terminals contain low to moderate densities of oxytocin receptors (OXTR) as studied with receptor autoradiography and experiments performed on mouse line with fluorescence-labeled OXTR (Yoshida et al., 2009; Froemke and Young, 2021). It is of interest that in the cerebral cortex the OXTRs appear to be found to a substantial degree on the inhibitory GABA interneurons showing somatostatin or parvalbumin immunoreactivities (Nakajima et al., 2014). A recent review has in fact been written on the role of OXT signaling at the synaptic connections (Bakos et al., 2018). OXTR exists at the presynaptic and postsynaptic levels of excitatory and inhibitory synapses modulating their synaptic transmission. OXT can improve postsynaptic and presynaptic glutamate transmission (Osako et al., 2001) and depress spontaneous GABA inhibitory postsynaptic currents involving a presynaptic mechanism. Furthermore, it is of interest that OXT *via* a certain class of prefrontal cortical interneurons can modulate female sociosexual behavior (Nakajima et al., 2014). It seems likely that the OXTRs modulation of brain networks takes place mainly through volume transmission which is true for other neuropeptides in the brain, including galanin and neuropeptide Y (Fuxe et al., 2010b; Borroto-Escuela et al., 2015a). However, it is also possible that the oxytocin GPCR heterocomplexes formed, while mainly located in extra-synaptic membranes, can also exist in synaptic membranes.

The major change in evolution of mammals, regarding the hormonal role of the OXT-OXTR system, may have been the appearance of an increasing number of oxytocin axon collaterals that are formed from hypothalamic neurons that innervate large parts of the brain. In this way, neuroendocrine information can reach large parts of the central nervous system (Jurek and Neumann, 2018) including the spinal cord and through the formation of heterocomplexes that integrate this information into the brain networks involved, especially for social behavior, food intake, and neuroendocrine events. See also the excellent review of Froemke and Young, which discusses the most recent findings on the neurocircuitry of oxytocin mainly in the context of social behavior (Froemke and Young, 2021). This integrative mechanism may be disturbed in mental diseases like depression and anxiety (Perez de la Mora et al., 2022).

Recently, the crystal structure of the human OXTR was established in a complex with the OXTR antagonist retosiban which is in clinical use for counteraction of labor (Waltenspuhl et al., 2020). It is of interest that the OXTR has a dependence on allosteric modulators like cholesterol and magnesium (Mg^2+^) for full activation (Klein et al., 1995a,b; Perez de la Mora et al., 2022). In the crystal structure, it was possible to observe that the cholesterol was bound in a pocket produced by transmembrane helices IV and V (Waltenspuhl et al., 2020). This feature gives a structural foundation for understanding how levels of cholesterol can allosterically influence the overall function of the OXTR (Waltenspuhl et al., 2020). In addition, the existence of two conserved residues with negative charges of transmembrane helices I and II of the OXTR were also identified (Waltenspuhl et al., 2020). These residues can be a site for the Mg^2+^ *via* electrostatic interactions, which may allow the development of allosterically enhanced agonist binding. This has provided a structural basis for the allosteric modulation produced by cholesterol and Mg^2+^ in the OXTR, which may be true also for other GPCRs, especially the vasopressin receptor and is poised to have important functional consequences for receptor functioning. Similarly, this concept has relevance for the allosteric receptor-receptor interactions in OXTR and other types of heteroreceptor complexes (Borroto-Escuela et al., 2012).

## Oxytocin Heteroreceptor Complexes

**Figure 1:**
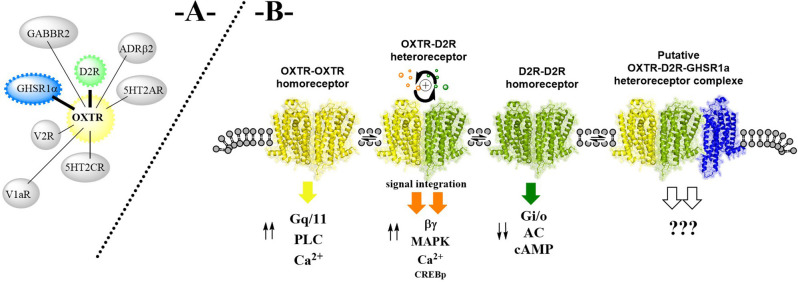
**(A)** On the existence of oxytocin (OXTR) heteroreceptor complexes (e.g., D2R-OXTR, GHS-R1a-OXTR, OXTR-5HT2AR, and OXTR-5HT2C, for further details see https://www.gpcr-hetnet.com/) in the plasma membrane of brain regions and their putative balance with higher order oxytocin heteroreceptor complexes through a more general participation of the ghrelin receptor protomers in the heterodimers shown. **(B)** As an example, the balance between an OXTR homodimer, a D2R homodimer, and an OXTR-D2R heterodimer is given in the plasma membrane together with the potential formation of an OXTR-D2R-GHSR1a heterocomplex. OXTR homodimer signaling involves an increase in phospho lipase C (PLC) activity and calcium levels (Ca^2+^) over Gq/11 activation while the D2R homodimer signaling takes place *via* activation of Gi/o producing inhibition of adenylate cyclase (AC) bringing down cyclic adenosine monophosphate (cAMP) formation. The ingration of these signals develops in the OXTR-D2R heterodimer and results in an enhanced activity over both the cAMP response element-binding protein (CREB), the mitogen-activated protein kinase (MAPK), and PLC-Ca^2+^ signaling pathways, mediated over the beta/gamma components of the G proteins.

**Figure 2:**
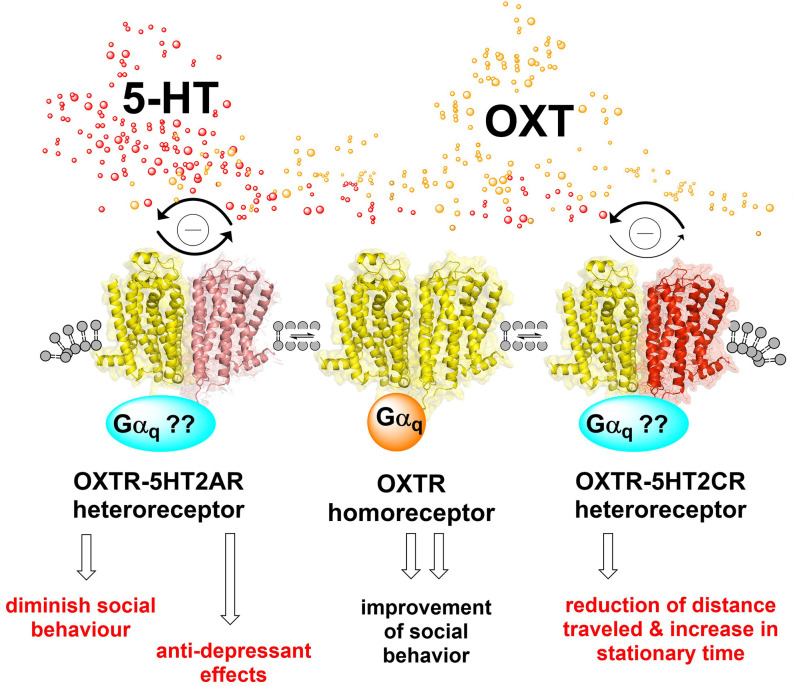
Oxytocin and serotonin heteroreceptor complexes in the brain. The OXTR-5-HT2AR heteroreceptor complex, shown as a heteroreceptor dimer in the plasma membrane, is found in the left region. The bidirectional antagonistic allosteric receptor-receptor interaction is outlined leading to attenuation of the oxytocin receptor protomer and 5-HT2AR protomer signaling as indicated by the connected two arrows in strong black color. A possible modulation in the G alpha q coupling of this heteroreceptor complex is indicated by the question mark. The 5HT2AR agonist-induced allosteric inhibition of the oxytocin receptor protomer can diminish its increase in social behavior (Froemke and Young, 2021). The Oxytocin receptor protomer (OXTR) can upon activation allosterically inhibit 5-HT2AR protomer signaling and diminish the 5-HT2AR signaling with possible antidepressant effects since 5-HT2AR mediates depressive effects (Borroto-Escuela et al., 2021; Perez de la Mora et al., 2022). The oxytocin homo-receptor complex, shown as a homo dimer, is found in the center of the illustration, coupled to G alpha q. It improves upon the activation of social behavior (Froemke and Young, 2021). The OXTR-5-HT2CR heteroreceptor complex, shown as a heteroreceptor dimer in the plasma membrane, is illustrated on the right side of the figure. The ability of the 5-HT2CR protomer (red) activation to allosterically attenuate the oxytocin receptor protomer G alpha q mediated signaling is substantially stronger (thick arrow) than the ability of the OXTR receptor agonist to allosterically diminish the 5-HT2CR G alpha q signaling (narrow arrow). It may involve differential changes in the degree of alterations in G alpha q coupling and of coupling also to other G proteins. In line with the biochemical results, the 5-HT2CR antagonist strongly enhanced the oxytocin receptor agonist-induced reduction of distance traveled and increase in stationary time (Chruścicka et al., 2021). These findings underline the functional relevance of the OXTR-5-HT2 complexes and their receptor-receptor interactions. Thus, the findings give indications that blockade of 5-HT2CR signaling can enhance oxytocin receptor signaling and produce improvement of social disease.

An extensive literature now supports the fact that Class A and B GPCRs function not only as monomeric entities but can crosstalk with other GPCRs and can even form dimers or higher-order oligomers (Terrillon and Bouvier, 2004; Borroto-Escuela et al., 2012, 2014; Schellekens et al., 2013a; Navarro et al., 2018; Milligan et al., 2019; Nemoto et al., 2022). In particular, the OXTR has been reported to be able to form homomers (Cottet et al., 2010; Busnelli et al., 2016) as well as to form dimers with other GPCRs, including with the highly related vasopressin V1a and V1b (Terrillon et al., 2003), the GHSR (Wallace Fitzsimons et al., 2019), the D2 (de la Mora et al., 2016), the 5HT2A (Chruścicka et al., 2019) and the 5HT2C (Chruścicka et al., 2021; Figures 1, 2). Interestingly, OXT was previously shown to interact with the orexigenic hormone ghrelin, where intravenous OXT administration reduced the circulating levels of ghrelin (Vila et al., 2009). In contrast, *in vitro* exposure of neurohypophyseal cells to ghrelin resulted in enhanced oxytocin secretion from the cells (Galfi et al., 2016). Previous data have also demonstrated that oxytocin administration modulates serotonin (5-HT) synthesis and 5-HT receptor binding potential, resulting in an overall modulation of the serotonergic system (Mottolese et al., 2014). The OXTR, GHSR, D2R, 5-HT2AR, 5-HT2CR, are known to play critical roles in a variety of physiological processes such as metabolism, and central appetite regulation, mood, and social behavior (Wardman et al., 2016; Grammatopoulos, 2017). Thus, OXTR crosstalk or dimerization will impact downstream signaling, conferring functional significance in several metabolic and stress-related disorders, including obesity and depression. Both, GHSR, 5HT2AR and 5HT2CR dimerization with the OXTR have been shown to result in attenuation of oxytocin-mediated downstream signaling (Figure 2; Chruścicka et al., 2019, 2021; Wallace Fitzsimons et al., 2019; Borroto-Escuela et al., 2021). However, the scale and functional outcome of OXTR, and other types of GPCR dimerization in the centrally regulated mechanisms are not yet fully appreciated and are only beginning to emerge.

### D2R-OXTR heterocomplexes

In 2013, evidence was obtained for the existence of D2R-OXTR heterocomplexes in the nucleus accumbens and the caudate putamen (Romero-Fernandez et al., 2013) using *in situ* proximity ligation assay (Borroto-Escuela et al., 2013). These results were validated by using the BRET technique in HEK293 cells after co-transfection with D2R^Rluc^ and OXTR^GFP2^ (de la Mora et al., 2016). In D2R binding saturation experiments in accumbal membranes using 3H-raclopride (D2R antagonist), oxytocin at 3 nM increased the maximal binding capacity (Bmax) values for the D2R antagonist, indicating that through allosteric receptor-receptor interactions, oxytocin can increase the availability of the D2R to bind the D2R antagonist due, e.g., to reduced internalization of the D2R protomer (Romero-Fernandez et al., 2013).

In 3H-raclopride competition experiments with dopamine (DA), oxytocin (3 nM) highly significantly increased the affinity of the high-affinity D2R agonist binding sites, giving indications that oxytocin can increase the D2R recognition and signaling in accumbal membranes (Romero-Fernandez et al., 2013). It was found that oxytocin increased D2R Gi/o coupling in accumbal membranes, using the GTP gamma S accumulation assay (Romero-Fernandez et al., 2013). Based on the outstanding work of Young and colleagues on the interactions between DA and OXT systems (Young and Wang, 2004) and in line with the results of Romero-Fernandez (Romero-Fernandez et al., 2013), it seems likely that the D2R-OXTR heterocomplexes play a significant role in social behavior. Based on the impressive study of Striepens et al. (2014); it becomes important to determine in future work if it is also possible to observe changes or not in 11C-raclopride binding assays in rodents. It is certainly of high interest to establish if there are differences in the ability of OXT to modulate D2Rs in, e.g., rodent vs. human.

It is of substantial interest that the enhanced bidirectional allosteric D2R-OXTR interactions (Figure 1) with improved signaling over the cyclic adenosine monophosphate (cAMP) response element-binding protein (CREB), mitogen-activated protein kinase (MAPK) and phospholipase C (PLC) signaling pathways, can have relevance for the anxiolytic effects observed upon microinjections of the D2R agonist quinpirole and oxytocin into the central amygdala (de la Mora et al., 2016). The Shock-probe burying test was used which represents an unconditioned model of anxiety/fear. The anxiolytic effects were blocked by co-infusion with a D2R-like antagonist raclopride in the central amygdala (de la Mora et al., 2016). Thus, restoring the faciliatory D2R-OXTR interactions can represent a new type of treatment for excessive anxiety. The receptor interface can involve transmembrane segment 5 (TM5) and the N-terminal region in view of the two triplet amino acid homologies observed in these regions that is hypothesized to favor the appearance of hot spots which increases the strength of the receptor interface formed (Tarakanov and Fuxe, 2010; Borroto-Escuela et al., 2018a).

It will be of interest to test if also putative D3R-OXTR complexes can be involved in social behavior in view of the existence of D2R-D3R heterocomplexes (Scarselli et al., 2001). D1R-D3R and A2AR-D3R heterocomplexes have also been demonstrated (Torvinen et al., 2005; Fiorentini et al., 2008) as well as D3R-nAChR heterocomplexes (Bono et al., 2020).

### Oxytocin receptor-ghrelin receptor (GHS-R1a) heterocomplexes

Over the last years, the ghrelin receptor (GHS-R1a) has been shown to heterodimerize with several other GPCRs, including the neurotensin receptor 1 (NTS1R; Takahashi et al., 2006), the dopamine D1R (Jiang et al., 2006; Schellekens et al., 2013a) and D2R (Kern et al., 2012); the melanocortin receptor 3 (MCR3; Rediger et al., 2011; Muller et al., 2013; Schellekens et al., 2013a) and the 5HT2CR (Schellekens et al., 2013a; Kern et al., 2015). For reviews on ghrelin and its receptor forming the mentioned heterocomplexes, see Schellekens et al. (2013b), Borroto-Escuela et al. (2014), Wellman and Abizaid (2015), and Ringuet et al. (2021). Similarly, the OXTR has also been shown to heterodimerize with the D2R (Romero-Fernandez et al., 2013; de la Mora et al., 2016). In addition, recent evidence from the Schellekens group has shown that the OXTRs can form functional heteroreceptor complexes with the GHS-R1a (Figure 1; Schellekens et al., 2013a,b) and also with the 5-HT2AR (Chruścicka et al., 2019) and 5-HT2CR (Schellekens et al., 2015), the latter two being discussed in later paragraphs. Notably, co-expression of the GHS-R1a and the OXTR was shown to significantly impair oxytocin-mediated Gαq signaling (Wallace Fitzsimons et al., 2019). Ghrelin is a gut hormone (28-amino acid peptide) that can cross the blood-brain barrier and reach the CNS, where it activates the GHS-R1a, resulting in a significant enhancement of appetite, food intake, and modulation of food reward signaling (Schellekens et al., 2013a,b; Schellekens et al., 2013c). However, the role of the GHS-R1a in the brain as a target for ghrelin is not clear, since penetration into the brain by ghrelin mainly may take place *via* rapid sensing of circulating ghrelin by hypothalamic appetite-modifying neurons (Schaeffer et al., 2013) and over the median eminence open to the portal blood (Rodriguez et al., 2010). Moreover, the GHS-R1a possesses a high constitutive activity that reaches 50% of its maximal activity that can be modulated by dynamic expression and allosteric mechanisms in GPCR-GHS-R1a complexes (Holst et al., 2003; Petersen et al., 2009; Schellekens et al., 2013a,b; Perez de la Mora et al., 2022). The novel GHS-R1a/OXTR pair was revealed by the Schellekens group using *in vitro* approaches and a novel flow cytometry-based fluorescence resonance energy transfer (fc-FRET) technique (Wallace Fitzsimons et al., 2019). Furthermore, co-location of the two receptor protomers was observed in primary hippocampal and hypothalamic cultures of postnatal day 1 (P1) Sprague Dawley rats. Wallace Fitzsimons et al. (2019) showed increased trafficking of the OXTR/GHS-R1a pair following co-expression (Chruścicka et al., 2018). Notably, the most significant finding was that the formation of the GHS-R1a-OXTR heterocomplex led to an attenuation of oxytocin-induced calcium mobilization mediated *via* G alpha q signaling. This reduction was diminished upon administration of a GHS-R1a antagonist (Wallace Fitzsimons et al., 2019). The mechanism was proposed to involve a slower recycling of the OXTR protomer in this heterocomplex and/or a partial switch in the OXTR signaling from G alpha q towards G alpha i signaling with inhibition of adenylyl cyclase (AC), cAMP, and protein kinase A (PKA) signaling pathway (Wallace Fitzsimons et al., 2019).

The latter mechanism is attractive and is in line with evidence that allosteric transmembrane receptor-receptor interactions have a significant role in altering the signaling of the participating receptor protomers (Fuxe and Borroto-Escuela, 2018; Borroto-Escuela et al., 2021). However, it remains to be determined if the GHS-R1a modulates the OXTR protomer recognition *via* allosteric receptor-receptor interactions and what the functional consequences are of this intricate interaction *in vivo*. As mentioned above, the GHS-R1a also forms heterocomplexes with other GPCRs like dopamine and serotonin receptor subtypes (Schellekens et al., 2013a, b). This raises the possibility of trimeric OXTR-GHS-R1a-D2R heterocomplexes, in addition to heteromeric complexes of GHS-R1a-OXTR, D2R-OXTR, and D2R-GHS-R1a, which may be independent of ghrelin for its signaling (Figure 1; Holst et al., 2003; Petersen et al., 2009). Therefore, It seems possible that the GHS-R1a can act as a dynamic adapter GPCR to modulate and fine-tune higher order OXTR heteroreceptor complexes across a wide range of functionalities, from social behavior, food intake to mood and reward functions (Schellekens et al., 2013b,c). Further mechanistic understanding as well as translation of heteromeric significance *in vivo* will be key for the full understanding of how the GHS-R1a operates in the OXTR heteroreceptor complexes of the brain.

### Hypothesis on the integrative operation of the D2R, OXTR, and GHS-R1a protomers forming multiple heterodimers and higher order heterocomplexes modulating food intake considering their molecular integration in the different circuits of food intake

A highly significant review has been written by Onaka and Takayanagi (2019) on the role of the oxytocin-OXTR system in food intake. Food intake activates vagal afferents which reach the nucleus tractus solitarius where A2 noradrenergic neurons (Dahlstroem and Fuxe, 1964) project to the hypothalamus, especially those expressing prolactin-releasing peptide (Okuno et al., 2012), where the hypothalamic oxytocin neurons become stimulated (Okuno et al., 2012). The result was an OXTR-mediated reduction of food intake with the termination of the meal. Under special conditions, an increased food intake was found (Onaka and Takayanagi, 2019). However, the oxytocin neurons may not be the major direct target. Instead, primary targets may involve the Agouti-related peptide (AgRP)/neuropeptide Y (NPY) positive neurons in the arcuate nucleus with orexigenic effects and the proopiomelanocortin (POMC) positive arcuate neurons with anorexic effects which play an important role in food intake (Sohn, 2015). These two neuronal populations project to neurons in the paraventricular hypothalamic nucleus expressing melanocortin 4 (MC4), almost lacking the OXTRs (Onaka and Takayanagi, 2019). There also exists a neuronal D2R positive population in the arcuate nucleus of the hypothalamus (Romero-Fernandez et al., 2014) that can contribute to the ability of the ergot D2R agonist cabergoline to inhibit food intake (Kern et al., 2015).

**Figure 3:**
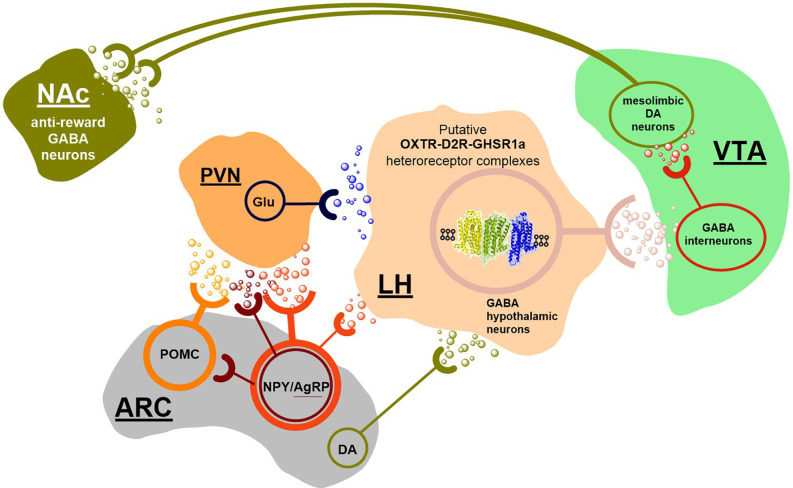
Hypothesis on the brain circuitry of food reward. Food intake activates vagal afferents which reach the nucleus tractus solitarius where A2-noradrenergic positive neurons project to the hypothalamus, where the hypothalamic oxytocin neurons become stimulated with reduction of food intake and termination of the meal (not shown). However, under certain conditions the oxytocin neurons may not be the major direct target. Instead, primary targets may involve the Agouti related peptide (AgRP)/neuropeptide Y (NPY) neurons in the arcuate nucleus with orexigenic effects and the arcuate proopiomelanocortin (POMC) neurons with anorexic effects which play an important role in food intake. These two neuronal populations project to neurons in the paraventricular hypothalamic nucleus expressing melanocortin 4 receptor, almost lacking the OXTRs. There also exists a neuronal D2R in the arcuate nucleus of the hypothalamus that can contribute to the ability of the ergot D2R agonist cabergoline to inhibit food intake. To understand how the various orexigenic and anorexigenic signals in the hypothalamus, including the gastric peptide ghrelin, can be integrated into their modulation of reward circuits involving the hedonic aspects of food intake, it is proposed the existence of a GABAergic neuronal population in the lateral hypothalamus. This population can form a nucleus that can receive these signals directly or indirectly. and integrate them mainly through two types of heteroreceptor complexes, OXTR-D2R-GHS-R1a high order heterocomplexes and NMDAR-D2R heterocomplexes. This Integration process can have a major role in determining the activity of these inhibitory GABA hypothalamic neurons. These key integrative GABA neurons send projections to the GABA interneurons inhibiting DA cell groups in the ventral tegmental area belonging to the meso-limbic DA reward neurons projecting to the nucleus accumbens. The major integrative mechanism in the key hypothalamic GABA neurons shown can be the postulated OXTR-D2R-GHSRIa high-order heteroreceptor complexes in the plasma membrane of extrasynaptic and synaptic membranes of these GABA neuron populations forming a GABA nucleus. This integrative mechanism can have a major role in controlling and modulating the glutamate drive on these GABA neurons by the ability of the activated D2R protomer to open the G protein-coupled inwardly rectifying potassium (GIRK) channels leading to hyperpolarization and reduction of the glutamate drive. The modulation of the glutamate drive can also involve NMDAR-D2R heterocomplexes. A dynamic balance between glutamate drive and D2R protomer mediated inhibition of these key GABA neurons can in this way be obtained with an appropriate firing of these inhibitory GABA neurons projecting onto the GABA interneurons in the VTA area. In this way, a suitable GABA release and correct inhibition of the meso-limbic DA reward neurons can be obtained. As a result, the GABA anti-reward neurons in the nucleus accumbens involved in food reward regulation can be properly regulated. This hypothesis will be tested in future work.

**Figure 4:**
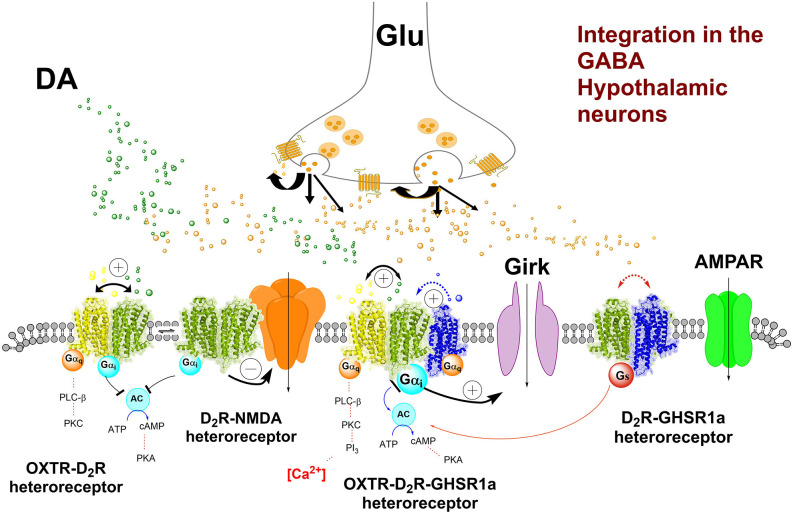
Hypothesis on the key heteroreceptor complexes in the proposed inhibitory GABA nerve cell population (nucleus) in the lateral hypothalamus projecting to the inhibitory GABA interneurons in the ventral tegmental area (VTA) controlling meso-limbic DA reward neurons in the VTA. The key higher order heterocomplexes OXTR-D2R-GHS-R1a and the NMDAR-D2R are shown in the plasma membrane of the GABA neurons. The trimeric complex is indicated to be in balance with the OXTR-D2R, OXTR-GHS-R1a, and D2R-GHS-R1a in the plasma membrane. The pharmacology indicates that D2R activation results in the inhibition of food intake related to the inhibition of food reward. The mechanism existing in the trimeric complex may be the D2R-induced opening of the GIRK channels leading to inhibition of the firing of the hypothalamic GABA neurons. Through positive allosteric receptor-receptor interactions the OXTR protomer can enhance the inhibitory D2R signaling. Also, GHS-R1a likely plays a role in this trimeric complex since the removal of the Ghrelin receptor blocks the inhibitions of food intake by the D2R protomer (see text). Thus, it seems likely that the existence of the Ghrelin receptor protomer is necessary for the correct allosteric receptor-receptor interactions to develop by allowing the appropriate conformational changes to develop in this trimericheteroreceptor complex. Thus, the D2R protomer activation can allow the inhibition of the hypothalamic GABA neurons projecting to the VTA GABA interneurons. Also, the potential existence of the NMDAR-D2R hetero complexes in the hypothalamic GABA neurons may help reduce the glutamate drive through D2R protomer-mediated inhibition of the NMDA receptors Thus, this mechanism may also contribute to lowering food-reward. There can exist other types of glutamate receptors located on the hypothalamic GABA neurons that can play an important role like the AMPA receptor and kainate receptors. They can contribute to strong glutamate activation of the hypothalamic. GABA neurons lead to considerable increase of glutamate drive and increased activity in the meso-limbic DA reward neurons by the inhibition of the GABA interneurons and increased food reward can develop also in this way.

To understand how all these transmitter signals can be integrated in their modulation of hedonic food intake, the existence of a GABAergic neuronal cell population is proposed in the hypothalamus forming a nucleus in which all these transmitter signals can be integrated to obtain a proper regulation of food reward (Figure 3). The GABA axons can then project to GABA interneurons inhibiting the meso-limbic DA reward neurons with origin in the ventral tegmental area (VTA) where several DA nerve cell subgroups exist (Figure 3; Dahlstroem and Fuxe, 1964; Fuxe, 1965a; Anden et al., 1966; Arbuthnott et al., 1970; Fuxe et al., 2010b). The GABA projection neurons will upon activation reduce the activity of the GABAergic interneurons and set free the activity of certain populations of VTA DA reward neurons, since their inhibitory synaptic GABA A receptor-mediated transmission becomes reduced (Figure 3; Suyama and Yada, 2018). As an example, we will focus on the relevance of the integrative allosteric D2R, OXTR, and GHS-R1a interactions in heteroreceptor complexes (Figure 4) altering the firing of the postulated hypothalamic GABA projections to the GABA interneurons reducing their inhibitory activity setting free activity in many meso-limbic DA reward neurons. Upon their activation DA can be released in the nucleus accumbens and activate D2R on the ventral striatal-pallidal GABA neurons producing inhibition of the activity of these GABA anti-reward neurons (Borroto-Escuela et al., 2018b). In this way, the palatable food intake can cause rewards to develop in the cortical networks (Schellekens et al., 2013a,b,c; Onaka and Takayanagi, 2019).

Coming back to the postulated hypothalamic GABA neurons potentially located within the lateral part of the medial forebrain bundle and projecting to certain GABA interneurons of the ventral tegmental area, it is proposed that they are enriched in D2 receptors like the ventral striatal-pallidal GABA anti-reward neurons (Borroto-Escuela et al., 2018b). It may be that these D2Rs are the major targets for the ability of the D2R agonist cabergoline to reduce food intake (Kern et al., 2015). In view of the demonstration of D2R-OXTR heterocomplexes with enhancing allosteric receptor-receptor interactions in the brain (Romero-Fernandez et al., 2013), it is suggested that they also exist in these postulated hypothalamic GABA neurons. Oxytocin has also been reported to preferentially bring down carbohydrate intake and leave fat intake unaffected (Onaka and Takayanagi, 2019). It can involve an allosteric enhancement of D2R protomer signaling in the D2R-OXTR heterocomplex. The major action of these events may be a hyperpolarization of these GABA neurons due to the D2R protomer-induced opening of the G protein inwardly rectifying potassium channels (GIRK channels; Figure 4), causing a reduction in the firing of these postulated GABA hypothalamic-VTA projection neurons setting to a substantial degree the GABA inter-neurons free from inhibition. Thus, due to an enhancement of the GABA interneuron-mediated inhibition of some VTA DA cell groups, some meso-limbic DA reward neurons can become inhibited and mediate the inhibition of food reward.

It should also be considered that the OXTR can form heterocomplexes with both the GHS-R1a and the D2R. Thus, a formation of the putative D2R-GHS-R1a-OXTR heterocomplex may exist and modulate the activity of the postulated hypothalamic GABA projection neurons to the GABA interneurons of the VTA (Figures 3, 4; Kern et al., 2012, 2015). When the ghrelin receptor is pharmacologically antagonized or knocked out, the D2R agonist cabergoline can no longer inhibit food intake. It is likely that it leads the allosteric modulation by the ghrelin receptor protomer of the D2R and OXTR protomers in the complexes is lost. It can lead to failure to reduce the firing of the hypothalamic GABA neurons and maintain inhibition of GABA interneurons. As a result, increased activity can develop in some meso-limbic DA reward neurons (Kern et al., 2012; Wellman and Abizaid, 2015). Going back to the key hypothalamic GABA neurons postulated to exist (see above), it seems clear that the D2R protomer-mediated inhibition of food intake depends also on the Ghrelin receptor protomer. The postulated key hypothalamic GABA neurons discussed, can thus contain D2R-GHS-R1a-OXTR heterocomplexes in which dynamic allosteric receptor-receptor interactions can develop to modulate the D2R signaling and enable dynamic changes in food reward. As mentioned, GHS-R1a-OXTR heterocomplexes have been demonstrated with the ability to modulate oxytocin signaling (Wallace Fitzsimons et al., 2019) and can exist in the GABA neurons postulated above.

However, as proposed also other hypothalamic orexigenic and anorexigenic transmitter signals may reach including these postulated key hypothalamic integrative GABA neurons indirectly or directly. It can involve the AgRP and NPY released from axons originating in the arcuate nucleus with orexigenic effects and POMC also from the arcuate nucleus with anorexigenic effects. They are regarded to mainly project to hypothalamic paraventricular neurons expressing MC4Rs (Onaka and Takayanagi, 2019). These neurons may project directly to the postulated key hypothalamic integrative GABA neurons and are glutamatergic (Onaka and Takayanagi, 2019). Their detailed connections have not been established.

The hypothesis introduced proposes that food reward regulation can be accomplished through the existence of key integrative GABA neurons in the lateral hypothalamus sending projections to GABA interneurons inhibiting DA subgroups in the VTA being part of the meso-limbic DA reward neurons to the nucleus accumbens and limbic cortex. Major integrative mechanisms in these key hypothalamic GABA neurons can be the high-order D2R-GHS-R1a-OXTR heterocomplexes in the plasma membrane of extra-synaptic and synaptic membranes and the integration of the synaptic glutamate receptors (Suyama and Yada, 2018) with the D2R, especially through NMDAR-D2R heterocomplexes (Liu et al., 2006). In such ways a dynamic balance between glutamate drive and D2R-mediated inhibition of these GABA neurons can be obtained, leading to an appropriate firing of the inhibitory GABA signaling onto GABA VTA interneurons obtaining a suitable GABA release and correct inhibition or lack of inhibition of the component of the mesolimbic DA neurons involved in food reward.

### A novel OXTR-5HT2AR-heterocomplex and its potential function for brain and behavior

The 5HT2AR-OXTR heteroreceptor complex was recently discovered in the laboratory of Dr. Schellekens (Chruścicka et al., 2019). Initial considerations for a potential dimer between 5-HT2AR and OXTRs were based on the fact that both GPCRs are located in 5-HT neurons and 5HT2ARs stimulate oxytocin release from the paraventricular hypothalamic neurons (Zhang et al., 2002; Yoshida et al., 2009). Therefore, a specific crosstalk between the OXTR and the 5-HT2AR receptors exists and the OXTR had also already been demonstrated in another heterocomplex of D2R-OXTR (Romero-Fernandez et al., 2013; Figures 1, 2). In the Schellekens lab, the first evidence for a physiological interaction came again from fc-FRET-based experiments using HEK293 cells (Chruścicka et al., 2019). A robust fc-FRET, depicted as a percentage of cells and median fluorescence was shown. It should be noted that the fc-FRET signal was not caused by overexpression, random collision, or dimerization of fluorescent proteins. Next, cellular colocalization of the OXTR and the 5-HT2AR was found not only in the plasma membrane but also intracellularly which is in line with a co-trafficking of both receptors together. Final evidence was delivered using *in situ* proximity ligation assay (PLA), which demonstrated the relevance of the 5-HT2AR-OXTR heterocomplexes *in vivo* in rodent brains.

High densities of PLA puncta were observed in the pyramidal cell layer of CA2 and CA3 in the rat dorsal hippocampus using confocal microscopy. Substantial densities of PLA puncta were also observed in the cingulate cortex around pyramidal nerve cell bodies and along apical dendrites. In the nucleus accumbens shell also accumulations of PLA puncta with high and low densities were found indicating that the 5-HT2AR-OXTR heterocomplex was found in certain types of nerve cell populations (Chruścicka et al., 2019). While no other brain regions have been investigated to date, these results are promising for the potential relevance of the 5HT2AR/OXTR pair in other regions of the brain as well.

Functionally, an attenuation of the Gαq-mediated OXTR and 5-HT2AR receptor protomer signaling was observed, upon the formation of this heteroreceptor complex (Chruścicka et al., 2019). The results indicate that this attenuation is dependent on the specific bidirectional antagonistic receptor-receptor interaction in this complex and not on their expression level, nor on the fluorescent tags or gene delivery mode. It should be underlined that none of the receptor antagonists for the two receptor protomers of the 5-HT2AR-OXTR heterocomplex blocked their signaling. Thus, the mechanism involved is not yet fully clear, but it may be that the orthosteric binding sites for the endogenous transmitter 5-HT and oxytocin remain in the heteroreceptor complex but not for the orthosteric antagonist binding site for the OXTR and 5HT2AR protomers in the 5-HT2AR-OXTR heterocomplex. This complex may offer new strategies for the treatment of mental diseases, especially depression (Perez de la Mora et al., 2022), and social behavior (Figure 2). It will be interesting to investigate if the GHS-R1a is also a potential heteromeric partner in this novel complex, forming a higher-order GHS-R1a/OXTR/5-HT1AR complex.

### A novel OXTR-5-HT2CR heterocomplex and its potential function for brain and behavior

The Schellekens group also recently demonstrated the formation of a 5-HT2CR-OXTR heterocomplex, using *in vitro* heterologous cell expression systems and fc-FRET combined with *ex vivo* proximity ligation assay (Chruścicka et al., 2021).

Noteworthy, co-expression of the 5-HT2CR protomer was able to markedly diminish the oxytocin-induced increases in Gαq-mediated calcium mobilization of the OXTR. The mechanism is the production of a constitutive allosteric brake on the G alpha q signaling in the OXTR protomer made possible through the formation of the 5-HT2CR-OXTR heterocomplex. Interestingly, the inhibition on OXTR mediated signaling was restored following a pharmacological targeting of the 5-HT2CR using RS102221 a specific 5-HT2CR antagonist. This suggests that the receptor interface mediating the allosteric wave requires an intact 5-HT2CR agonist binding site for the allosteric wave to pass into the OXTR protomer from the 5-HT2CR protomer. A similar but weaker antagonist allosteric event developed when 5-HT activated the 5-HT2CR protomer in the presence of the OXTR protomer forming the heteroreceptor complex (Figure 2). Thus, also a constitutive allosteric wave-induced by the OXTR, although reduced compared to the wave induced by 5-HT2AR, exists to bring down the 5-HT2CR G alpha q signaling upon activation by 5-HT. In line with these results, it was found in cells co-expressing the two receptor protomers that the basal internalization and trafficking of the OXTR protomer was enhanced, an action which was again diminished by the 5-HT2CR antagonist (Chruścicka et al., 2021).

Using a proximity ligation assay, the 5-HT2CR-OXTR heterocomplexes were clearly present in brain slices in the dorsal hippocampus and the retro-splenial granular and agranular cortex. They were mainly found in the pyramidal cell layer of the CA1, CA2, and CA3 regions with the highest density in the CA3 region. These regions are mainly built up of glutamate neurons. PLA signals were also found over some GABA interneurons mainly scattered in the cell layers of oriens and radiatum, especially of the CA1 region. It will be of high interest to study in detail many other relevant regions like the forebrain, the caudate putamen and nucleus accumbens, the hypothalamus, and the serotonin, dopamine, and noradrenaline nerve cell groups of the lower brain stem. It also will be of high interest to compare the PLA positive distribution pattern of the 5-HT2AR-OXTR and the 5-HT2CR-OXTR heterocomplexes in adjacent sections, e.g., dorsal hippocampus and nucleus accumbens *ex vivo*.

It was previously demonstrated that OXTR and 5-HT2CR have the ability to participate in locomotor activity (Nebuka et al., 2020). It, therefore, became of interest to test if also the 5-HT2CR-OXTR heterocomplexes have a role in locomotor activity (Chruścicka et al., 2021). It was of substantial interest that a 5-HT2CR antagonist enhanced the hypoactivity induced in mice by oxytocin (Figure 2). The 5-HT2CR antagonist used was SB242084 and the specificity of the effect observed was indicated by the observation that this 5-HT2CR antagonist alone increased locomotor activity (Chruścicka et al., 2021). It will be of interest to determine the cellular location and functions of the 5-HT2CR-OXTR heterocomplex involved, once the 5-HT2CR-OXTR heterocomplexes have been mapped out using the PLA method. However, the basal ganglia is also a putative target (Chruścicka et al., 2021).

In relation to functional relevance, both the OXTR and 5-HT2CR are expressed in dorsal raphe neurons with their interactions leading to diminished anxiety (Yoshida et al., 2009). It will therefore be interesting to demonstrate if targeting the OXTR/5HT2C dimer has a potential anxiolytic effect.

## Understanding Tuning (Modulation) and Fine-Tuning (Metamodulation) Neurotransmission in The Brain in A Novel Way. The Role of The Heteroreceptor Complexes

These concepts have been discussed over many years and especially by the Ribeiro and Sebastiao group (Ribeiro and Sebastiao, 2010) in relation to adenosine and its adenosine receptors. Based on the current work on OXT and OXTR heterocomplexes and other types of heteroreceptor complexes, it is proposed that the GPCR heterocomplexes are important mediators of meta-modulation. The activation, e.g., of the OXTR by OXT leads to modulation of OXTR protomer signaling, and through the allosteric receptor-receptor interactions induced by the activated OXTR protomer, other protomers of distinct OXTR heteroreceptor complexes undergo meta-modulation (fine-tuning), including D2R, 5-HT1AR, and 5-HT2CR protomers (Romero-Fernandez et al., 2013; Chruścicka et al., 2019, 2021). This molecular integrative mechanism is hypothesized to play a major role in metamodulation both at the postsynaptic and presynaptic level and in extrasynaptic membrane regions where heteroreceptor complexes have a major integrative role (Fuxe et al., 2010a; Borroto-Escuela et al., 2017).

## Limitations and Future Studies

While GPCRs are mainly described as functioning monomers, the formation of GPCR heterocomplexes is increasingly being recognized and has important consequences for the discovery and development of GPCR-based pharmaceutical targets as heteromerization is associated with altered GPCR signaling (Borroto-Escuela et al., 2017, 2018b; Fuxe and Borroto-Escuela, 2018).

Several techniques have been successfully employed to demonstrate the OXTR-GPCR interactions, such as *in vitro* cellular overexpression systems, where colocalization has been measured with fluorescence microscopy, receptor dimerization with fc-FRET, BRET, as well as using *ex vivo* PLA and immunofluorescence (Schellekens et al., 2015; de la Mora et al., 2016; Chruścicka et al., 2019; Wallace Fitzsimons et al., 2019; Chruścicka et al., 2021). The pharmacological relevance of interactions has been equally studied using downstream analysis, including calcium mobilization assays and cAMP assays. In addition, single cell co-expression analysis using mouse and human cortical data from the Allen Brain Atlas has brought some translational validation of potential GPCR co-expression. Computational approaches for modeling and predicting GPCR dimerization have been provided by the GGIP web server (Nemoto et al., 2022) and online resources including the GPCR-HetNet (Borroto-Escuela et al., 2014). The relevance of GPCR heterodimerization *in vivo* has been discussed in the past. Furthermore, functional GPCR interactions have revealed physical heteroreceptor complexes with allosteric receptor-receptor interactions between receptor pairs, with each complex resulting in unique alterations to GPCR recognition, signaling, and trafficking (Fuxe and Borroto-Escuela, 2016, 2018). Nevertheless, a gap in the physiological relevance of many heteroreceptor complexes remains, with a limited number of *in vivo* heterodimerization studies published compared to the number of identified heteroreceptor complexes. Future studies should focus on the validation of heteroreceptor complex formation *in vivo* under healthy and pathophysiological conditions, as well as in depth analysis of the heterodimer’s physiological role.

## Future Aspects

The OXTR has a high potential to form heteroreceptor complexes, the same is true for GHS-R1a including the GHS-R1a-OXTR heterocomplexes as demonstrated over the last two decades. In 2019 the GHS-R1a-OXTR heteromerization was found for the first time (Wallace Fitzsimons et al., 2019). It will now be of highest importance to determine if in fact in general the OXTR and GHS-R1a come together in the brain forming high-order heteroreceptor complexes also including the DA, noradrenaline (NA), and 5-HT receptor subtypes, and other types of GPCRs. It seems possible that also, e.g., receptor tyrosine kinase (RTK) and ionotropic receptors can be involved in the complexes formed. Based on the role of the OXTR and GHS-R1a, especially in the central autonomic and neuroendocrine system, social behavior and food intake, these integrative complexes can have a special role in the limbic system, the hypothalamus, and the lower brain stem.

Evidence suggests that oxytocin can increase the density and length of 5-HT axons during development, which indicates the involvement of RTK in the 5-HT and oxytocin receptor-receptor interactions (Eaton et al., 2012). There are also signs of support for the view that 5-HT and oxytocin interplay in the nucleus accumbens significantly mediate the rewarding aspects of social behavior (Dolen et al., 2013).

It is also of interest that galanin-like peptides can be released by stress from neurons in the arcuate nucleus and intracerebroventricular injections of galanin-like peptides enhance the release of oxytocin (Onaka et al., 2005). Furthermore, galanin receptors have been found in the arcuate nucleus of the hypothalamus (Kinney et al., 1998; Leibowitz, 1998) and oxytocin receptors are widespread throughout the hypothalamus (Yoshimura et al., 1993). These observations open the possibility that galanin receptors may form complexes with oxytocin receptors, especially in the hypothalamus with a focus on the arcuate nucleus, which should be tested in future work.

The OXTR and GHS-R1a may usually come together as heterodimer complexes in which the high constitutional activity of the GHS-R1a may alter the conformation of the OXTR and facilitate its affinity and binding to other receptors like the various monoamine receptors. However, it should also be noted that the GHS-R1a also may form complexes with other receptors like monoamine GPCRs. Such interactions may also facilitate the formation of high-order heteroreceptor complexes. An alternative is also that GHS-R1a *via* its interaction with some surrounding receptors can reduce their affinity for the OXTR and block the formation of unsuitable high-order heteroreceptor complexes.

This can become an exciting new field for understanding integrative processes in the plasma membrane. However, increases in our understanding of the molecular hot spots in the receptor interface involving the postulated key role of the trimeric amino acid homologies in the receptor interface formed, especially in transmembrane domains (Tarakanov and Fuxe, 2010; Borroto-Escuela et al., 2018a) as well as electrostatic interactions, especially in intracellular domains, is warranted before the field will be able to fully interrogate the functional significance of, e.g., OXTR heteromerization (Woods et al., 2005).

## Author Contributions

We confirm and declare that all authors meet the criteria for authorship according to the ICMJE, including approval of the final manuscript, and they take public responsibility for the work and have full confidence in the accuracy and integrity of the work of other group authors. They have substantially contributed to the conception or design of the current *Review Article*. They have also participated in the acquisition, analysis, and interpretation of data for the current manuscript version. Furthermore, they have helped revising it critically for important intellectual content; and final approval of the version to be published. In addition, they have contributed to this last version of the manuscript in writing assistance, technical editing, and language editing. DB-E, HS, MP, and KF have a prominent role in the conception of the hypothesis introduced and their discussion. All authors contributed to the article and approved the submitted version.
